# Functional modulation of the human voltage-gated sodium channel Na_V_1.8 by auxiliary β subunits

**DOI:** 10.1080/19336950.2020.1860399

**Published:** 2020-12-29

**Authors:** S. T. Nevin, N. Lawrence, A. Nicke, R. J. Lewis, D. J. Adams

**Affiliations:** a School of Biomedical Sciences and the Institute for Molecular Bioscience, The University of Queensland, Brisbane, Australia; b Institute for Molecular Bioscience, The University of Queensland, Brisbane, Australia; c Illawarra Health and Medical Research Institute (IHMRI), University of Wollongong, Wollongong, Australia

**Keywords:** Na_v_, β subunits, voltage-gated sodium channel, recovery, inactivation, *Xenopus* oocytes, membrane expression, chimeric β subunits

## Abstract

The voltage-gated sodium channel Na_v_1.8 mediates the tetrodotoxin-resistant (TTX-R) Na^+^ current in nociceptive primary sensory neurons, which has an important role in the transmission of painful stimuli. Here, we describe the functional modulation of the human Na_v_1.8 α-subunit in *Xenopus* oocytes by auxiliary β subunits. We found that the β3 subunit down-regulated the maximal Na^+^ current amplitude and decelerated recovery from inactivation of hNa_v_1.8, whereas the β1 and β2 subunits had no such effects. The specific regulation of Na_v_1.8 by the β3 subunit constitutes a potential novel regulatory mechanism of the TTX-R Na^+^ current in primary sensory neurons with potential implications in chronic pain states. In particular, neuropathic pain states are characterized by a down-regulation of Na_v_1.8 accompanied by increased expression of the β3 subunit. Our results suggest that these two phenomena may be correlated, and that increased levels of the β3 subunit may directly contribute to the down-regulation of Na_v_1.8. To determine which domain of the β3 subunit is responsible for the specific regulation of hNa_v_1.8, we created chimeras of the β1 and β3 subunits and co-expressed them with the hNa_v_1.8 α-subunit in *Xenopus* oocytes. The intracellular domain of the β3 subunit was shown to be responsible for the down-regulation of maximal Na_v_1.8 current amplitudes. In contrast, the extracellular domain mediated the effect of the β3 subunit on hNa_v_1.8 recovery kinetics.

## Introduction

Voltage-gated sodium channels (VGSCs), which mediate the rising phase of the action potential in excitable cells, consist of a 260 kD pore-forming α subunit of which there are nine mammalian subtypes known (Na_v_1.1-Na_v_1.9), each with distinct tissue distribution and biophysical properties. The α subunits associate with one or more auxiliary β subunits of which there are four known subtypes: β1 (36 kD), β2 (33 kD) [1,2], β3 [3] and β4 (38 kD) [4]. The α subunits consist of four domains, each containing six transmembrane helices flanked by intracellular N- and C-termini; whereas the β subunits all adopt the immunoglobulin-like fold with an intracellular C-terminus, one α-helical membrane-spanning domain and two extracellular β-sheets [5–7]. Although the α subunit alone is sufficient for the formation of a functional channel pore, the β subunits are required for the physiological kinetics and voltage-dependent gating observed in native cells [8].

Primary sensory neurons (dorsal root, nodose and trigeminal ganglion neurons) express a multitude of VGSC α subunit subtypes [9–13] including Na_v_1.1, 1.2, 1.6, 1.7, 1.8 and 1.9 as well as all four β subunits [3,4,14]. Chronic pain states of both inflammatory and neuropathic origin are characterized by changes in the expression profile of VGSCs in sensory neurons, which in turn leads to altered neuronal excitability. In particular, the VGSC subtype Na_v_1.8 plays a major role in pain, as demonstrated in Na_v_1.8 knockout mice, which display attenuated pain behavior in comparison to wild-type mice [15]. Na_v_1.8 is now considered to have key roles in both inflammatory and neuropathic pain [16], and gain-of-function point mutations in Na_v_1.8 have been reported in humans with painful neuropathy [17]. Furthermore, Na_v_1.8 is thought to be the most important VGSC for low temperature-induced pain [18]. Inhibition of Na_v_1.8 can reduce inflammatory and/or neuropathic pain in animal models [19], demonstrating a key role for this VGSC α subunit in pain states. Na_v_1.8 is selectively expressed by small dorsal root ganglion (DRG) neurons involved in nociception [12], and mediates a slowly-inactivating tetrodotoxin-resistant (TTX-R) Na^+^ current which is up-regulated in inflammatory pain states. In contrast, Na_v_1.8 is down-regulated in neuropathic pain but still considered important in influencing neuronal excitability [20–24]. These changes are partially attributed to alterations in the levels of growth factors that regulate channel expression, however, other mechanisms, such as altered modulation by auxiliary β subunits, may also be involved [25,26]. β subunits can significantly modulate the properties of VGSC α subunits by regulating kinetics and voltage-dependence of gating, regulating cell surface expression levels and act as adhesion molecules (see review [16]). In both rat [5,27] and human DRG [28], neuropathic pain is characterized by an increase in immunoreactivity for the β3 subunit. The β3 subunit co-localizes with Na_v_1.8 in sensory neurons [29], suggesting that an up-regulation of the β3 subunit may affect the activity and biophysical properties of Na_v_1.8.

The expression of rat Na_v_1.8 (rNa_v_1.8) in *Xenopus* oocytes [5,30] and mammalian cells [31–33] has been described previously, and modulation of rNa_v_1.8 by auxiliary β subunits has been characterized [5,30]. The expression of human Na_v_1.8 (hNa_v_1.8) in *Xenopus* oocytes [34], human embryonic kidney (HEK293) cells [35], and mammalian sensory neuron-derived ND7/23 cells [36] has also been reported previously. Although some aspects of β subunit modulation of hNa_v_1.8 have been described previously in mammalian cells [37], others remain to be characterized, such as β subunit-mediated effects on recovery from inactivation. A previous study examined how β1 affected repriming of rNa_v_1.8 [30] but the functional modulation of hNa_v_1.8 by β subunits expressed in *Xenopus* oocytes has not been characterized. The *Xenopus* oocyte expression system is useful for screening ion channel targeting compounds [38]. When screening for compounds that can inhibit or modulate hNa_v_1.8, this α subunit should ideally be expressed together with β subunits to better mimic the *in vivo* situation. Thus, it is important to evaluate how β subunits modulate hNa_v_1.8 in the *Xenopus* oocyte expression system.

In the present study, we describe the modulation of hNa_v_1.8 by auxiliary β subunits in *Xenopus* oocytes. We found that β3 affected the maximal current amplitude and recovery from inactivation whereas the β1 and β2 subunits had little influence on these parameters. Both the extracellular [4,39] and intracellular domains of β subunits can interact with the VGSC α subunit [40,41]. To investigate which domains of the β3 subunit mediated these specific effects, we also studied the modulation of hNa_v_1.8 current amplitude and repriming by chimeric β1/β3 and β3/β1subunits.

## Materials and methods

### cRNA preparation

Rat β1 and β2 subunits were gifts (Dr A.L. Goldin, UC Irvine, CA). Rat β3 cDNA was cloned by RT-PCR, subcloned into the pNKS2 oocyte expression vector [42] and C-terminally fused to a hexahistidine tag. Constructs encoding human Na_v_1.8 (cloned into pcDNA3.1), rat Na_v_1.2 (cloned into pLCT1) rat β1, rat β2, rat β3, rat β3[L8F, R20S, F174L,V210A] as well as rat β1/β3 and β3/β1 chimeras (all in pNKS2) were linearized and cRNA was synthesized using SP6 or T7 *in vitro* transcription kits (Ambion mMessage mMachine, Austin, TX) as described previously [34].

### Oocyte preparation and microinjection

*Xenopus* oocytes were defolliculated with collagenase (Type I, Sigma) at 3 mg/ml in OR-2 medium that contained (mM): 82.5 NaCl, 2 KCl, 1 MgCl_2_, 5 HEPES-NaOH, pH 7.4 for 2–3 hours at room temperature. Oocytes were stored at 18°C in sterile ND96 medium containing (mM): 96 NaCl, 2 KCl, 1.8 CaCl_2_, 5 HEPES-NaOH, pH 7.4 supplemented with 5 mM pyruvate and 50 µg/ml gentamycin. Glass pipettes for microinjection were pulled from glass capillaries (3–000-203 GX, Drummond Scientific Co., Broomall, PA). The cRNAs were diluted in water to 0.5 μg/μl, and then diluted further to the appropriate concentrations to inject a total of 2.5 ng of RNA for the hNa_v_1.8 α subunit, alone or in combination with 0.5–5 ng RNA for the β subunits as outlined for each experiment. 50 nL RNA was injected into each oocyte using a microinjector (Nanojet II, Drummond Scientific Co.).

### Analysis of expression and glycosylation status of the β3 subunit

*Xenopus laevis* oocytes were injected with 50 nl aliquots of β3 cRNA (0.5 mg/ml). For metabolic labeling of total protein, oocytes were incubated overnight at 19°C with L-[^35^S]-methionine at ~100 Mbq/ml with ~ 0.2 MBq/oocyte (Amersham) in sterile ND96 (96 mM NaCl, 2 mM KCl, 1 mM CaCl_2_, 1 mM MgCl_2_ and 5 mM HEPES, pH 7.4). For selective labeling of β3 protein at the plasma membrane, oocytes were cultured for three days after cRNA injection. Intact oocytes were then treated with [^125^I]-sulfo-SHPP (Amersham), a membrane impermeable derivative of the Bolton-Hunter reagent. Sulfo-SHPP (Pierce) was radioiodinated as described previously [43]. At ambient temperature the following reagents were rapidly and subsequently added to 0.5 μg sulfo-SHPP in 2 μl DMSO: 18.5 MBq of carrier-free Na^125^I, 10 μl 0.5% chloramine T in 0.5 M sodium phosphate buffer pH 7.5, 100 μl 0.1% DL-α-hydroxyphenyl acetic acid in 0.1 M NaCl, and 10 μl 1.2% sodium metabisulfite in 0.05 M sodium phosphate buffer pH 7.5. 30 μl aliquots of this mixture were immediately added per 10–12 oocytes. After 60 min incubation on ice with occasional gentle mixing, oocytes were washed in ND96 and His-tagged protein was purified via Ni^2+^-NTA agarose beads (Qiagen) as described previously [43] using 0.5% n-dodecyl-β-D-maltoside (ULTROL Grade, Calbiochem-Novabiochem GmbH, Bad Soden, Germany) as detergent. Shortly, oocytes were homogenized in 0.1 M phosphate buffer (20 μl per oocyte) containing 0.4 mM Pefabloc® SC (Fluka, Buchs, Switzerland) and 0.5% n-dodecyl-β-D-maltoside (ULTROL Grade, Calbiochem-Novabiochem GmbH). The homogenate was incubated on ice for 15 min and the extract was then cleared by centrifugation (10 min at 15,000 rpm in a desktop centrifuge). 100 μl of the clear supernatant were diluted with 400 μl of the above buffer and supplemented with 30 μl Ni^2+^-NTA agarose beads and 10 mM imidazole. After 30 min of incubation under continuous inversion, the agarose-bound protein was washed four times with 1 ml phosphate buffer containing 0.1% dodecyl maltoside, 0.4 mM Pefabloc® SC, and 25 mM imidazole. Subsequently, protein was eluted from the agarose beads with non-denaturing elution buffer (20 mM Tris-HCl, 100 mM imidazole-HCl, 10 mM EDTA and 0.5% dodecyl maltoside, pH 7.8). Purified protein was kept at 0°C until analyzed. 10 μl aliquots of protein were supplemented with SDS sample buffer and separated on 10% polyacrylamide gels. Gels were dried and exposed to BioMax MR films (Kodak) at – 80°C. For analysis of the glycosylation status, 10 ml aliquots of purified protein were supplemented with reducing (20 mM DTT) SDS sample buffer and 1% octylglucoside (Calbiochem-Novabiochem GmbH) and incubated for 1 h at 37°C with 0.5 or 5 IUB milliunits endoglycosidase H (Endo H) or 5 IUB milliunits PNGase F (New England Biolabs GmbH, Frankfurt, Germany).

### Construction of the β subunit chimeras

A *Bsm I* site was introduced at position 654 of the rat β3 sequence (in pNKS2) using site-directed mutagenesis (QuikChange® II XL, Stratagene, CA). Rat β1 (with naturally occurring *Bsm I* site at position 805) and rat β3 pNKS2 constructs were digested with *Bsm I* and either 5ʹ or 3ʹ vector restriction sites to excise the intracellular or extracellular β subunit fragments respectively. Digested fragments were gel purified using a QIAquick gel extraction kit (QIAGEN, Germany) and ligated with T4 DNA Ligase (Fermentas) to obtain the β1/β3 and β3/β1 chimeras. Chimera junctions were checked for correct β subunit switching by DNA sequencing.

### Electrophysiological Recording of Na^+^ currents

Whole cell depolarization-activated currents mediated by hNa_v_1.8 or rNa_v_1.2 were recorded from *Xenopus* oocytes 3 days after cRNA injection using the two-electrode (virtual ground circuit) voltage clamp technique. Oocytes were placed in a (~400 µl) bath containing the appropriate recording solution mounted on the stage of a dissecting microscope, impaled with glass electrodes and voltage-clamped using a GeneClamp 500B amplifier (Axon Instruments) or an OpusXpress work station (Molecular Devices, Union City, CA). Microelectrodes were pulled from borosilicate glass (GC150TF, Harvard Apparatus) and typically had resistances of 0.3–1.5 MΩ when filled with 3 M KCl. All recordings were made at room temperature (20–23°C). During recordings, oocytes were perfused continuously at a rate of ~1.5 ml/min. Voltage-steps were generated using pCLAMP8 or OpusXpress software (Molecular Devices). Data were low pass filtered at 1 kHz, digitized at 10 kHz, and leak-subtracted on-line using a – P/6 protocol and analyzed off-line. Tetrodotoxin (TTX) was applied via a gravity-fed perfusion system. For each experiment, at least 3 different batches of oocytes were used.

### Data analysis

All data were analyzed using Clampfit 8 software (Molecular Devices) and graphs and curves were constructed and analyzed using GraphPad Prism 4.0 (San Diego, CA). Mathematical formulas are described below. All statistical analyses was performed using one-way ANOVA with Tukey’s multiple comparison test, or, when indicated in the figure legend, two-tailed T-test.

The voltage-dependence of activation was determined by measuring the amplitude of the Na^+^ current elicited by depolarization to various membrane potentials. Voltage-dependent Na^+^ conductance *(G)* was determined from transformations of current-voltage relationship (*I–V*) curves using the formula:

(1) $$G=\;\left[I/\left(V-V_{r}\right)\right]$$

where *I* is peak current amplitude, *V* is the test membrane potential, and *V*_r_ is the measured or extrapolated reversal potential. Current activation curves were fitted with a sigmoidal Boltzmann function that identifies the voltage at which the VGSC is half-maximally activated:

(2) $$G/G_{0}=\left[1/\left(1+exp\left(V_{0.5}-\;V\right)/K_{v}\right)\right]$$

where *G* represents the conductance at various membrane potentials, G_0_ is peak conductance, *V_0.5_* is the voltage where the VGSCs are half-maximally activated, *V* is the depolarized membrane potential and *K_v_* is the slope constant.

Steady-state inactivation at various membrane potentials was determined by applying 1s pre-pulses to different voltages ranging from – 120 mV to 0 mV immediately followed by a test pulse to the membrane potential generating peak Na^+^ current. The Na^+^ current amplitude elicited by the test pulse was normalized to the amplitude elicited after a pre-pulse to – 120 mV, where steady-state inactivation is minimal. Inactivation curves were fitted with a single Boltzmann function:

(3) $$I/I_{0}=\left[1/\left(1+exp\left(V_{0.5}-\;V\right)/K_{v}\right)\right]$$

where *I*/*I_0_* represents the fraction of current available, *V_0.5_* is the voltage where the VGSCs are half-maximal inactivated, *V* is the depolarized membrane potential and *K_v_* is the slope constant.

To determine recovery from inactivation of VGSCs, *Xenopus* oocytes were depolarized to 0 mV for 1 s to inactivate VGSCs and allowed different time periods (2.5 ms – 1 s) to recover at – 70 mV before a depolarizing pulse was applied to generate peak Na^+^ current. The Na^+^ current elicited by this pulse (*I*) was normalized to the current amplitude of an identical pulse not preceded by an inactivating pulse (*I_0_*). The fraction of Na^+^ current recovered was plotted against recovery time and fitted with single or multiple exponential equations of the form:

(4) $$I/I_{0}=\;1\;-\;\left[exp\;\left(-t/\tau_{1}\right)\right]$$

(5) $$I/I_{0}=\;1-\left[F1\cdot \;exp\;\left(-t/\tau_{1}\right)\;+F2\cdot \;exp\;\left(-t/\tau_{2}\right)\right]$$

where *I*/*I_0_* represents the fraction of recovered current; *t* represents the recovery time; *F1* and *F2* represent the fractions of current recovering with the time constants *τ*_1_ and *τ*_2._

The time constants for current inactivation were determined by fitting a single or double exponential function to the decay phase of the current:

(6) $$I/I_{0}=\;1\;-\;\left[exp\;\left(-t/\tau_{1}\right)\right]$$

(7) $$I/I_{0}=\;1-\left[F1\cdot \exp\;\left(-t/\tau_{1}\right)\;+F2\cdot \exp\;\left(-t/\tau_{2}\right)\right]$$

where *I*/*I_0_* represents the fraction of current remaining, *t* represents time and *τ_1_* (and *τ_2_*) the time constant (s) for inactivation. *F1* and *F2* represent the fraction of current in phase when the decay curve was fitted with a double exponential function. In contrast to the other curves which were analyzed by GraphPad Prism 4.0, this analysis was performed directly in ClampFit 8.

## Results

### Functional expression of human of Na_v_1.2 and Na_v_1.8 in Xenopus oocytes

**Figure 1.**
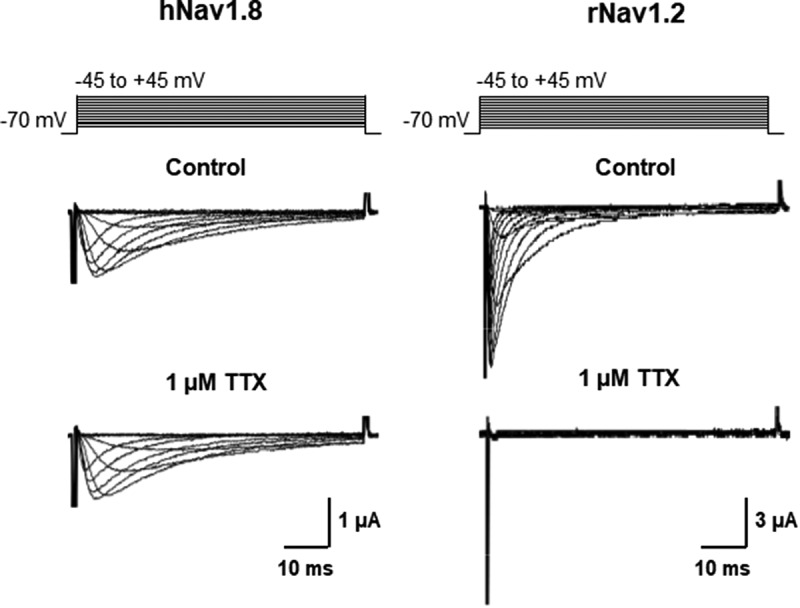
Expression of human Na_v_1.8 and rat Na_v_1.2 in *Xenopus* oocytes. (a) When expressed in *Xenopus* oocytes, hNa_v_1.8 mediates an inward Na^+^ current with slow activation and inactivation kinetics that is unaffected by 1 μM tetrodotoxin (TTX). (b) In contrast, rat Na_v_1.2 mediates a Na^+^ current exhibiting fast activation and inactivation kinetics that is completely abolished by the application of 1 μM TTX. Oocytes were held at – 70 mV and depolarized to voltages between – 50 and +40 mV in 10 mV increments. External solutions containing TTX (1 µM) were applied through the perfusion system

When expressed in *Xenopus* oocytes, hNa_v_1.8 produced a slowly-inactivating depolarization-activated Na^+^ current, which was not affected by 1 µM TTX (Figure 1), as has been shown previously [33]. The TTX-S VGSC subtype rNa_v_1.2 was expressed for comparison. In contrast to hNa_v_1.8, the Na^+^ current mediated by rNa_v_1.2 exhibited fast activation and inactivation kinetics and was completely blocked by 1 µM TTX (Figure 1). In the absence of β subunits, the voltage-dependence of activation and inactivation were both best fitted with single Boltzmann functions. Half-maximal activation (V_0.5_) was determined to be – 2.9 ± 0.4 mV (n = 56), whereas half-maximal inactivation occurred at – 43.5 ± 0.7 mV (n = 40) (Table 1). Recovery from inactivation consisted of two phases with distinct time constants (Table 1).

**Table 1.** Effects of the β1, β2 and β3 subunits on biophysical properties of hNa_v_1.8

Gating and current decay				
	Na_v_1.8	Na_v_1.8 + β1	Na_v_1.8 + β2	Na_v_1.8 + β3
V_0.5 (activation)_ (mV)	–2.9 ± 0.4 (56)	–9.8 ± 0.5*** (31)	–3.5 ± 0.8 (25)	–2.0 ± 0.6 (35)
V_0.5 (inactivation)_ (mV)	–43.5 ± 0.7 (46)	–54.2 ± 1.1*** (20)	–42.7 ± 1.7 (20)	–42.4 ± 1.4 (20)
τ_decay_ (ms)	9.1 ± 0.2 (40)	5.6 ± 0.9* (24)	8.8 ± 0.6 (12)	8.1 ± 1.6 (32)
Recovery from inactivation				
	Na_v_1.8	Na_v_1.8 + β1	Na_v_1.8 + β2	Na_v_1.8 + β3
τ_1_ (ms)	6.7 ± 0.9	7.0 ± 1.2	5.5 ± 0.5	14.1 ± 3.0**
τ_2_ (ms)	62.5 ± 4.0	67.9 ± 5.0	59.9 ± 2.5	331.5 ± 29.5 ****
_%_ fast	46.3 ± 2.9	43.6 ± 3.4	47.6 ± 1.8	24.8 ± 1.7****
n	18	15	15	21

Data given as mean ± SEM (n = number of oocytes). *p ≤ 0.05, **p ≤ 0.01, ***p ≤ 0.001,

****p ≤ 0.0001.

### Biochemical confirmation of synthesis and surface expression of the β3 subunit

**Figure 2.**
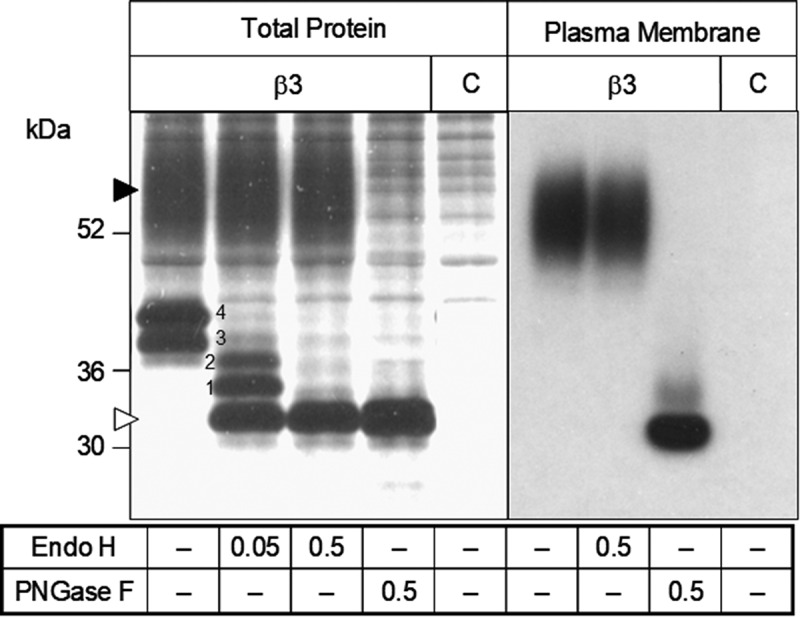
Biochemical analysis of the synthesis and plasma membrane transport of the sodium channel β3 subunit in *Xenopus laevis* oocytes. Oocytes injected with cRNA encoding the His-tagged β3 subunit or non-injected controls (c) were metabolically labeled with [^35^S]-methionine (left panel) or surface-iodinated with [^125^I]-sulfo-SHPP (right panel). His-tagged protein was purified via Ni^2+^-NTA-agarose, treated with endoglycosidases (concentrations given in IUB milliunits/ml sample) as indicated, and separated on a 10% SDS-PAGE gel. Black and white triangles indicate complex glycosylated and completely deglycosylated protein, respectively. Numbers 1–4 indicate the Endo H-sensitive core-glycosylated and partly deglycosylated forms of the protein

The rat β1 and β2 subunits had previously been shown to express and functionally modulate VGSCs in *Xenopus* oocytes [44]. To demonstrate that the β3 subunit was expressed and localized to the plasma membrane, oocytes were injected with a cRNA encoding a histidine (His)-tagged β3 subunit (β3-His) and metabolically labeled total β3-His protein as well as the selectively radio-iodinated membrane fraction of β3-His were purified via Ni-NTA agarose and analyzed by SDS-PAGE with and without prior endoglycosidase treatment. The β3 subunit was efficiently expressed in the plasma membrane and showed a uniform band of complex glycosylated protein, even in the absence of an α-subunit (Figure 2). Deglycosylation with PNGase revealed the predicted size of ~30 kDa whereas partial deglycosylation with Endo H confirmed the efficient complex glycosylation and revealed the four extracellular N-linked glycosylation sites.

### Effects of β subunits on the current amplitude of hNa_v_1.8

To first confirm that the β subunits exerted the expected effects upon expression in *Xenopus* oocytes, their modulation of Na_v_1.2 was first investigated. As shown previously [44,45], the β1 and β3 subunits shifted voltage-dependence of inactivation in the hyperpolarising direction and accelerated current decay kinetics of rNa_v_1.2, whereas the β2 subunit was without effect (Supplementary Fig. 1).

**Figure 3.**
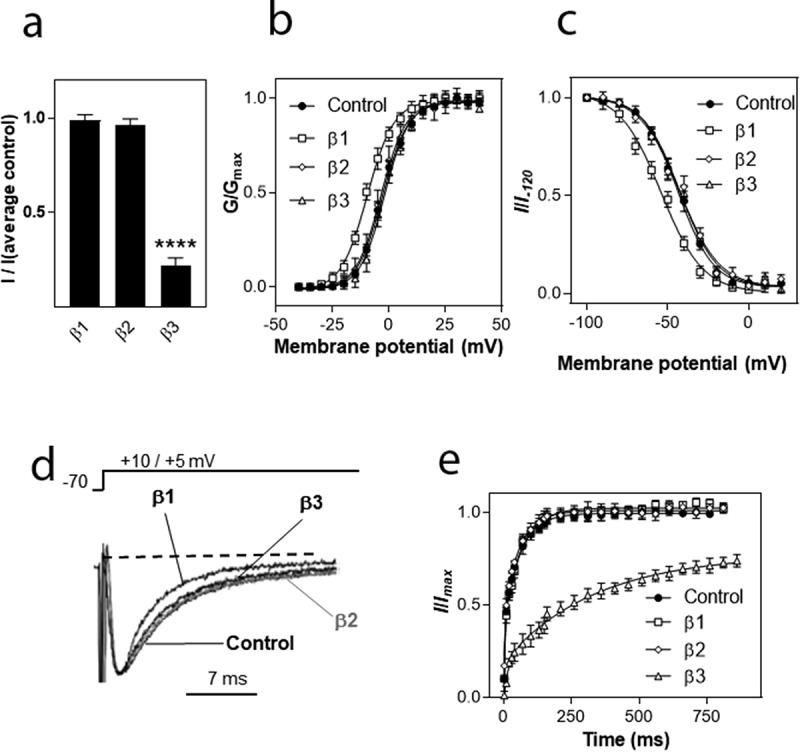
Modulation of hNa_v_1.8 by auxiliary β subunits. (a) Effects of β subunits on Na^+^ current amplitude. *I* represents maximal Na^+^ current amplitude of oocytes expressing hNa_v_1.8 (2 ng cRNA/oocyte) alone or in combination with β1, β2 or β3 (5 ng cRNA/oocyte). *I*(average control) represents the average maximal Na^+^ current amplitude of oocytes expressing only hNa_v_1.8. Maximal Na^+^ current amplitude was determined by step depolarizations to voltages between – 50 and +50 mV (5 mV increments) from a holding potential of – 70 mV. The voltages at which maximal Na^+^ current amplitude was obtained was +5 mV for hNa_v_1.8 + β1 and +10 mV for the other combinations (including Na_v_1.8 in the absence of β subunits). Curves show the Na^+^ conductance (G) obtained at different voltages relative to the maximal conductance (G_max_). Conductance curves were fitted with single exponential functions for the hNa_v_1.8 α subunit alone and in the presence of the various β subunits. (c) Voltage-dependence of inactivation. *I* represents the Na^+^ current elicited by a depolarizing pulse to the voltage generating maximal Na^+^ current amplitude immediately after long (1 s) pre-pulses to different voltages. *I_–120_* represents the Na^+^ current amplitude elicited by an identical depolarizing pulse generated after a long pre-pulse to – 120 mV, where inactivation is minimal. *I*/*I_−120_* represents the fraction of maximal Na^+^ current available after steady-state inactivation at each voltage. (d) Inactivation kinetics. Superimposed traces normalized to the same value are shown for Na^+^ currents mediated by hNa_v_1.8 in the absence and presence of the β1, β2 and β3 subunit (5 ng cRNA/oocyte). Oocytes were held at – 70 mV and depolarized to the voltage that elicited maximal Na^+^ current amplitude. (e) Recovery from inactivation. The fraction of Na^+^ current recovering from steady-state inactivation after different periods of time (2.5 ms – 1 s) was determined for hNa_v_1.8 (2.5 ng cRNA/oocyte) expressed alone or together with the β1, β2 or β3 subunit (5 ng cRNA/oocyte). Na^+^ current was first inactivated by a 1 s pulse to 0 mV. After a variable recovery period ranging from 2.5 ms – 1 s, a depolarizing pulse to elicit maximal Na^+^ current amplitude was applied. The Na^+^ current amplitude after different recovery times (*I*) was compared to the Na^+^ current amplitude elicited by an identical control pulse that was not preceded by inactivation (*I_max_*). The recovered fraction of Na^+^ current (*I/I_max_*) was plotted against recovery time and fitted with double exponential functions

After establishing that the β subunits had the expected effects on rNa_v_1.2, we investigated their effect on hNa_v_1.8. Expression of hNa_v_1.8 (2 ng/oocyte) alone and with the β1, β2 or β3 subunits (5 ng cRNA/oocyte), corresponding to an α:β ratio of 1:2.5. The β1 and β2 subunits did not affect the Na^+^ current amplitude of hNa_v_1.8, however, the β3 subunit caused a pronounced decrease in maximal Na^+^ current amplitude (I_max_) to 22 ± 4% of control (n = 40; p ≤ 0.001) (oocytes expressing only hNa_v_1.8; n = 56; average Na^+^ current amplitude 0.79 ± 0.38 μA) (Figure 3(a)).

### Effects of β subunits on current kinetics/channel properties

We then determined the effects of the β subunits on the biophysical properties of hNa_v_1.8, again at an α:β ratio of 1:2.5 (Figure 3(b–e)). The β1 subunit caused a hyperpolarizing shift of 6.9 mV in the activation curve of hNa_v_1.8 (Figure 3(b)); the value for V_0.5_ (activation) was significantly different between these two groups (p ≤ 0.001; Table 1). The β1 subunit also significantly altered voltage-dependence of inactivation of hNa_v_1.8 by shifting V_0.5_ 10.68 mV in the hyperpolarizing direction (p ≤ 0.001; Figure 3(c) and Table 1). Furthermore, the β1 subunit accelerated the inactivation kinetics of hNa_v_1.8 (single exponential fits, p ≤ 0.1; Figure 3(d) and Table 1).

We next investigated whether the β subunits modulated recovery from inactivation (repriming). Recovery from inactivation of hNa_v_1.8 occurs in two phases, as has been reported previously [36]. The β3 subunit strongly decelerated recovery from inactivation whereas the other β subunits were without effect (Figure 3(e)). In the presence of the β3 subunit, both τ_1_ (τ_fast_) and τ_2_ (τ_slow_) were significantly slower than for hNa_v_1.8 expressed alone (p ≤ 0.01 and p ≤ 0.0001 for τ_1_ and τ_2_, respectively, Table 1). The percentage of current recovering with fast kinetics was also significantly lower in the presence of the β3 subunit than in the other three groups (p ≤ 0.0001; Table 1).

**Figure 4.**
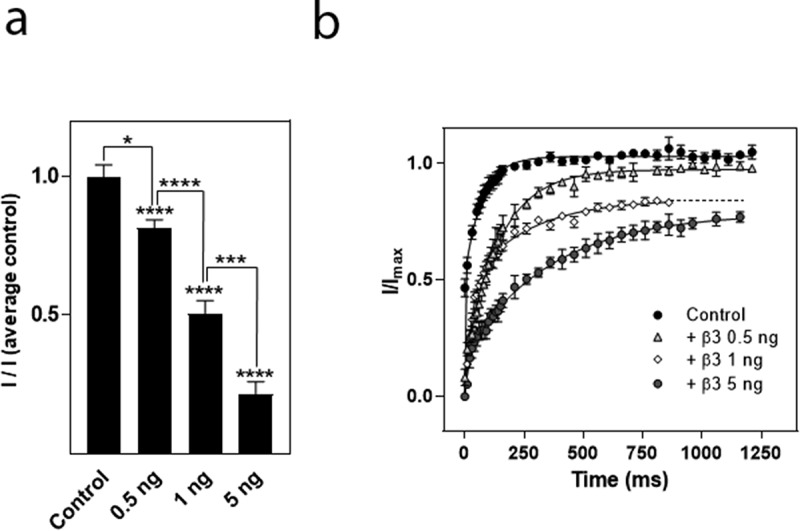
Effects of the α:β3 ratio on β3-mediated modulation of the hNa_v_1.8 current amplitude and recovery from inactivation. (a) Effects of the α:β3 ratio on current amplitude. Maximal Na^+^ current amplitude was recorded from oocytes injected with cRNA for hNa_v_1.8 (2.5 ng/oocyte) alone or together with 0.5, 1, or 5 ng of cRNA encoding the β3-subunit. *I* represents maximal Na^+^ current amplitude in the various groups while *I*(average control) represents the average maximal Na^+^ current amplitude of control (oocytes expressing only hNa_v_1.8). N = 30–41 oocytes/group. (b) Effects of the α:β3 ratio on the repriming kinetics of hNa_v_1.8. Recovery from inactivation was determined as described for hNa_v_1.8 alone. The Na^+^ current amplitude after different recovery times (*i*) was compared to the Na^+^ current amplitude generated by an identical control pulse (*I_max_*). The repriming curves were fitted with double exponential functions (N ≥ 10 oocytes/group)

To determine whether the effect of the β3 subunit on I_max_ of hNa_v_1.8 was dependent on the α:β3 ratio, different amounts of β3 cRNA (0.5, 1 or 5 ng) were injected into each oocyte in the presence of the same amount of hNa_v_1.8 cRNA (2.5 ng), corresponding to α:β3 ratios of 1:0.25, 1:0.4 or 1:25, respectively. We found that the effect of the β3 subunit on hNa_v_1.8 maximal current amplitude was dependent on the α:β3 ratio (Figure 4(a)). Similarly to the down-regulation of current amplitude by the β3 subunit, the effects on repriming were dependent on the α:β3 ratio (Figure 4(b)).

### Functional comparison of rat and human β3 subunits

**Figure 5.**
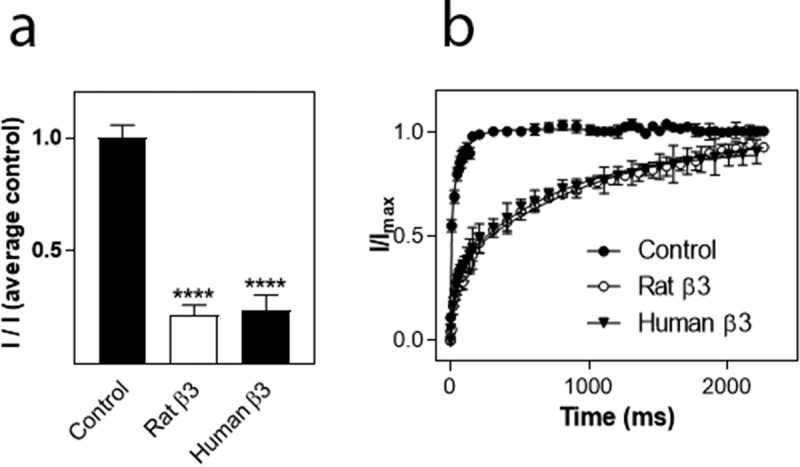
Comparison of modulation of hNa_v_1.8 by the rat and human β3 subunits. (a). Effects on Na^+^ current amplitude. Maximal Na^+^ current amplitude was determined for oocytes expressing hNa_v_1.8 alone or in combination with the rat or human β3-subunit. *I* represents the maximal Na^+^ current amplitude of oocytes expressing hNa_v_1.8 alone or in combination with the β3 subunit. I_(average control)_ represents the average maximal Na^+^ current amplitude of oocytes expressing only hNa_v_1.8. ****significantly different from control, p ≤ 0.0001. (b) Comparison of the modulation of recovery from inactivation of hNa_v_1.8 by the rat and human β3 subunits (N = 15–23 oocytes/group)

The β subunits used in this study were of rat origin. Rat and human β1 subunits share 93% homology in their amino acid sequence, rat/human β2 subunits share 96% homology and rat/human β3 subunits are 98% homologous. The high degree of homology suggests that the modulation of VGSC α-subunits does not differ between rat and human β subunits. The most striking modulation of hNa_v_1.8 was mediated by the β3 subunit. To verify that the human and rat β3 subunits mediated similar effects on hNa_v_1.8, the four amino acids of rat β3 that differ were mutated to the corresponding residues in the human β3 subunit (L8F, R20S, F174L and V210A). When co-expressed with hNa_v_1.8, rβ3[L8F, R20S, F174L, V210A] caused almost identical effects to wild-type rat β3 on current amplitude and recovery from inactivation of hNa_v_1.8 (Figure 5), suggesting that rat and human β3 modulate hNa_v_1.8 in a similar manner.

### Effects of auxiliary β1/β3 subunits chimeras on hNa_v_1.8

To investigate which part of the β3 subunit mediated the effects on hNa_v_1.8 current amplitude and recovery from inactivation, respectively, we created chimeras of the β1 and β3 subunit in which the extracellular domain of β1 was combined with the intracellular domain of β3 (β1_ext_/β3_int_) or vice versa (β3_ext_/β1_int_). The hNa_v_1.8 α subunit was then co-expressed with the chimeric β subunits in a 1:1 ratio. For this series of experiments, the current mediated by hNav1.8 expressed alone exhibited an I_max_ of 0.82 ± 0.04 µA (n = 172). As for the previous series of experiments, the β1 subunit had no significant effect on the I_max_ of Na_v_1.8, whereas co-expression with the β3 subunit caused a significant decrease (p < 0.001) of the Na_v_1.8 current amplitude (n > 9). The β1_ext_/β3_int_ chimera caused a similar reduction of hNa_v_1.8 I_max_ to that observed for the β3 subunit, whereas the β3_ext_/β1_int_ chimera subunit with hNa_v_1.8, did not significantly reduce I_max_ when compared to the hNa_v_1.8 control (Figure 4(a)). For these series of experiments, 2.5 ng of each cRNA was injected into each oocyte. Thus, the α:β3 ratio was 1:1 rather than 2:5, explaining why the amplitude was not reduced to the same extent as what is shown in Figure 3(a). Similarly, when hNa_v_1.8 was co-expressed with both β1 and β3, the ratio was 1:0.5:0.5, effectively diluting the reducing effect of the β3 subunit in comparison to the result shown in Figure 3(a).

**Figure 6.**
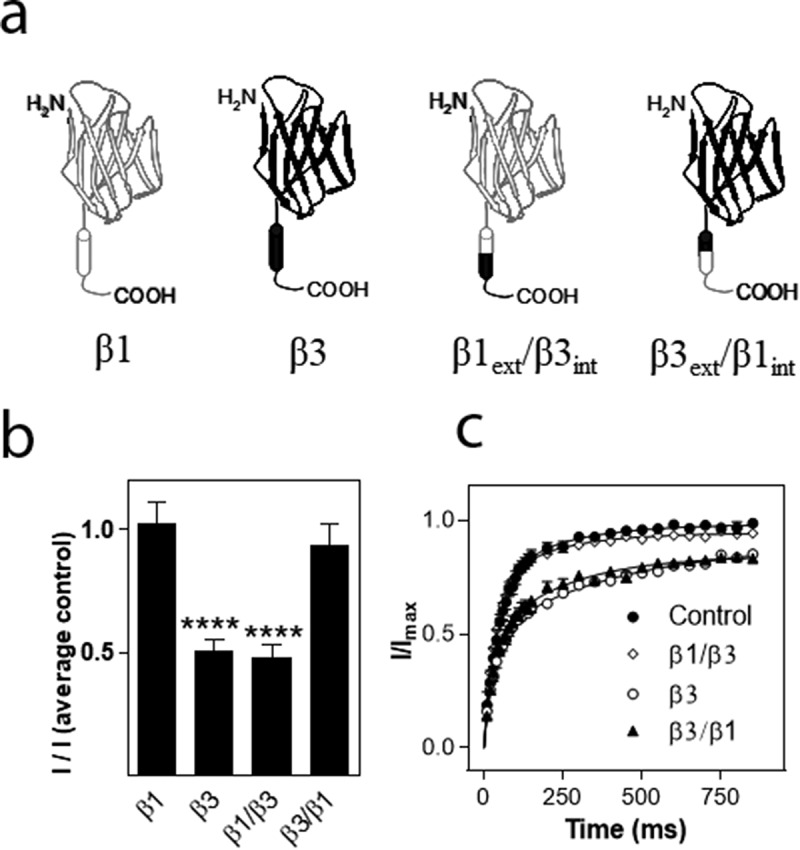
Effects of β subunits chimeras on maximal current amplitude and recovery from inactivation of hNa_v_1.8. (a). Schematic showing the structure of the wild-type β1 and β3 subunits and the constructed chimeras (β1: white, β3: black). (b) Effects on Na^+^ current amplitude. Maximal Na^+^ current amplitude was determined for oocytes expressing hNa_v_1.8 alone or in combination with the rat β3 chimera subunits. *I* represents maximal Na^+^ current amplitude of oocytes expressing hNa_v_1.8 alone or in combination with the β3 chimera subunits

We then assessed the effects of the chimeric β subunits on the recovery from inactivation of hNa_v_1.8. The β3 subunit as well as the β1_ext_/β3_int_ chimera significantly decelerated recovery from inactivation (p < 0.01) whereas the β3_ext_/β1_int_ chimera had no effect on recovery from inactivation (Figure 6(b)). Interestingly, whereas the β3 subunit decelerated τ of both the fast and the slow phase, the β1_ext_/β3_int_ chimera increased τ_2_ by approximately 30 ms (from 54 ± 5 ms to 88 ± 16 ms; n ≥ 20) but did not significantly alter τ_1_. Co-expression of hNa_v_1.8 with the β3 subunit or the β1_ext_/β3_int_ chimera only allowed 90% recovery of the maximum current obtained within a second, whereas full recovery was seen within 5 s of all the combinations used.

## Discussion

This study reports on the modulation of human Na_v_1.8 expressed in *Xenopus* oocytes by auxiliary β subunits. Na_v_1.8 is implicated in pain states and remains a promising target for drug discovery, for which the *Xenopus* oocyte expression system is a valuable screening platform [38]. To better mimic the natural environment in DRG neurons, Na_v_1.8 can be co-expressed with β subunits in *Xenopus* oocytes. Therefore, it is important to determine how β subunits modulate hNa_v_1.8 in this system.

When expressed in *Xenopus* oocytes, hNa_v_1.8 mediated a Na^+^ current with the TTX-resistance and slow kinetics characteristic of Na_v_1.8 in native DRG neurons [12,13] as has been shown previously [33]. The gating properties of hNa_v_1.8 in the absence of β subunits was similar to human Na_v_1.8 expressed in *Xenopus* oocytes [33], with minor differences from that previously reported for rat Na_v_1.8 [5,30]. Repriming kinetics were similar to that previously described for human Na_v_1.8 in mammalian cells [36]. The β1 subunit accelerated current decay kinetics for hNa_v_1.8, as has been described previously for rNa_v_1.8 [30,46]. β1 also caused hyperpolarizing shifts of both voltage-dependence of activation and inactivation of hNa_v_1.8. Similar shifts in voltage-dependence of activation and inactivation mediated by the β1 subunit have been reported for rNa_v_1.8 expressed in *Xenopus* oocytes [5,30,46] and mammalian cells [37]. In the present study, we did not find any effects of the β3 subunit on the voltage-dependence of activation/inactivation. This is consistent with data from rNa_v_1.8 in mammalian cells [37], however, β3 expressed with rNa_v_1.8 in *Xenopus* oocytes shifted both curves in the hyperpolarizing direction [47] or shifted the inactivation curve in the depolarizing direction [46].

The most pronounced effects of any of the auxiliary β subunits on hNa_v_1.8 were the modulation of recovery from inactivation and maximal Na^+^ current amplitude by the β3 subunit. β3 markedly decelerated the repriming kinetics of hNa_v_1.8 and reduced maximal Na^+^ current amplitude to ~25% of control levels (α:β3 ratio 1:2.5). This modulation of repriming resembled the effect of lidocaine and related compounds on VGSCs, including Na_v_1.8 [48]. Delayed recovery from inactivation mediated by the β subunits has not been reported previously. Instead, other studies have shown that the β3 subunit can accelerate repriming of VGSCs, Na_v_1.5 [49] and Na_v_1.3 [5,50]. Whilst we did not find that the β1 subunit modulated recovery from inactivation, a previous study has shown that β1 accelerated the first phase but decelerated the second phase of rat Na_v_1.8 repriming [30].

Unlike the β1 and β2 subunits, β3 has been shown to significantly reduce the current density of Na_v_1.8 [37]. This correlates well with our observation showing that β3 down-regulates the maximal current amplitude. However, two other studies report an up-regulation of rNa_v_1.8 current amplitude associated with β3 co-expression in *Xenopus* oocytes [5,46]. Differences in cRNA concentration and/or incubation time post-injection between studies may contribute to these differences, along with differences in human and rat Na_v_1.8, which are only 82% homologous. Furthermore, whilst we did not find that the β1 subunit affected the current amplitude of hNa_v_1.8, other studies on rNa_v_1.8 in *Xenopus* oocytes [46] and mammalian cells [37] have reported that β1 can increase current amplitude/current density. Again, discrepancies can be due to differences in the precise experimental design or expression of endogenous factors between expression systems. Therefore, it is a priority to evaluate the effect of the β subunits on Na_v_1.8 in native sensory neurons and a study of the expression of human and rat Na_v_1.8 in DRG neurons revealed subtle differences in the biophysical properties of the channels [51].

VGSC β subunits can function as cell adhesion molecules (CAMs), playing important roles in cell-cell adhesion. β subunits also modulate cell surface levels of VGSCs, most likely via anchoring the VGSC to the cytoskeleton [52,53]. Thus, the modulation of hNa_v_1.8 current amplitude by the β3 subunit may involve regulation by several mechanisms including modulation of channel opening probability, stabilization of the channel in the plasma membrane, cross-linking with other α or β subunits, alterations in trafficking or, less likely, signaling events leading to altered mRNA levels. Single-channel patch-clamp recording experiments in mammalian cells could be used to determine whether the β3 subunit directly alters the opening probability of hNa_v_1.8.

The VGSC is believed to exist *in vivo* as a heterodimer or heterotrimer consisting of one α subunit and one or two β subunits. Traditionally, it was considered that one α subunit can interact with one non-covalently linked (β1 or β3) and one disulfide-linked β subunit (β2 or β4) [2,54]. Recent studies suggest that interactions between VGSC α and β subunits are far more complex. One recent study, which investigated the structure of the immunoglobulin (Ig) domain of the β3 subunit using crystallography and single-molecule resolution imaging, reported that this domain assembles as a trimer. This study also reported that the β3 subunit can bind to multiple sites on the Na_v_1.5 α-subunit and induce the formation of α subunit oligomers, possibly resulting in cross-linking of multiple α and β subunits [41]. In the current study, we observed that the key effects of β3 appeared to be dependent on the α:β3 ratio (or the amount of β3 cRNA injected). It is possible that the number of β3 subunits in the membrane was not enough to saturate all Na_v_1.8 α subunits when the lower β3 cRNA concentrations were injected, however, more complex interactions such as β3 subunit crosslinking could also be involved. To date, we do not know whether such interactions modulate cell surface levels or biophysical properties of VGSCs.

It was previously thought that VGSC α and β subunits primarily interacted via their extracellular domains, however, more recent findings have demonstrated that their intracellular domains also interact. A recent study reported on the cryo-EM structure of the electric eel Na_v_1.4 α-subunit (EeNa_v_1.4) in complex with the β1 subunit. This study showed that the extracellular Ig domain of β1 docks with extracellular loop 5 (from domain I) and loop 6 (from domain IV) of the α-subunit, whereas the β1 transmembrane helix interacts with the third voltage-sensing domain (VSD_III_) of the α subunit [40]. Our data obtained using chimeras of the β1 and β3 subunits show that the extracellular domain of the β3 subunit mediated the effects on recovery from inactivation, whereas the down-regulation in current amplitude was modulated by the intracellular domain of the β3 subunit. Na_v_1.8 can interact with several intracellular proteins including cytoskeletal proteins, channel-associated proteins, motor proteins and enzymes which may regulate Na_v_1.8 membrane density [55]. In particular, annexin light chain (p11) is a strong regulator of trafficking and cell surface levels of Na_v_1.8 [56]. Ubiquitination and subsequent proteasomal degradation have also been shown to potently regulate cell surface levels of Na_v_1.8 [57]. It is thus possible that the β3 subunit interferes with another intracellular regulatory protein, indirectly modulating Na_v_1.8. In regard to recovery from inactivation, it is possible that interactions between the extracellular domains of Na_v_1.8 and the β3 subunit indirectly modulate sites in Na_v_1.8 involved in repriming, such as the transmembrane S6 segment [49] or the S3-S4 linker of domain IV [58].

All four known VGSC β subunits are expressed in sensory neurons [14,29,59]. The β3 subunit is the main β subunit expressed in nociceptive neurons and is therefore most likely to modulate VGSC behavior in these neurons [5,28,60]. Both the β1 and β3 subunits appear to play a role in pain, since they are up-regulated in rat and human DRG in neuropathic pain states [5,61]. The main role of the β1 and β3 subunits in alteration of DRG Na^+^ current profiles in neuropathic pain appears to be due to interactions with the VGSC subunit induced in neuropathic pain states. Heterologous expression experiments in *Xenopus* oocytes and mammalian cells have demonstrated that both the β1 and β3 subunits can further accelerate the already rapid repriming kinetics of Na_v_1.3, possibly promoting repetitive firing. In addition, β1 and β3 subunits lower the activation threshold of Na_v_1.3, thereby further contributing to increased excitability [5,50]. β3 has also been shown to co-localize with Na_v_1.7 in small dorsal root ganglion neurons and when co-expressed in mammalian cells, β3 modulated the gating properties of Na_v_1.7 with hyperpolarizing and depolarizing shifts in activation and inactivation, respectively. β3 also accelerated recovery from inactivation; together, these alterations may increase neuronal excitability [60].

The reduction of Na_v_1.8 current amplitude observed in the present study, consistent with a previous study in mammalian cells [37], would theoretically decrease the activity of Na_v_1.8 when translated into an *in vivo* situation. Furthermore, a decelerated recovery from inactivation could decrease the opening probability of the channel. The down-regulation in TTX-R Na^+^ current and the up-regulation of the β3 subunit in the DRG in neuropathic pain states are thought to be two independent phenomena that are caused by alterations in growth factor levels [62–65]. However, the data presented in the current and previous study [37] present a mechanism to explain how these two phenomena may be interrelated. If this is the case, the increased levels of the β3 subunit may contribute to the suppression of TTX-R Na^+^ current observed in neuropathic pain states.

## Supplementary Material

Supplemental Material

Supplemental Material
