# Supplementary material for: Functional modulation of the human voltage-gated sodium channel NaV1.8 by auxiliary β subunits
Source: Channels (Austin). 2020 Dec 29;15(1):79–93. doi: 10.1080/19336950.2020.1860399 (PMC7781643; doi:10.1080/19336950.2020.1860399)
Supplement: Supplemental Material [file KCHL_A_1860399_SM8675.docx]

**Table 1.** Effects of the β1, β2 and β3 subunits on biophysical properties of hNa_v_1.8

**Gating and current decay**

|  | **Na_v_1.8** | **Na_v_1.8 + β1** | **Na_v_1.8 + β2** | **Na_v_1.8 + β3** |
| --- | --- | --- | --- | --- |
| V_0.5 (activation)_  (mV) | −2.9 ± 0.4  (56) | −9.8 ± 0.5^***^  (31) | −3.5 ± 0.8  (25) | −2.0 ± 0.6  (35) |
| V_0.5 (inactivation)_  (mV) | −43.5 ± 0.7  (46) | −54.2 ± 1.1^***^  (20) | −42.7 ± 1.7  (20) | −42.4 ± 1.4  (20) |
| τ_decay_  (ms) | 9.1 ± 0.2  (40) | 5.6 ± 0.9^*^  (24) | 8.8 ± 0.6  (12) | 8.1 ± 1.6  (32) |

**Recovery from inactivation**

|  | **Na_v_1.8** | **Na_v_1.8 + β1** | **Na_v_1.8 + β2** | **Na_v_1.8 + β3** |
| --- | --- | --- | --- | --- |
| τ_1_ (ms) | 6.7 ± 0.9 | 7.0 ± 1.2 | 5.5 ± 0.5 | 14.1 ± 3.0^**^ |
| τ_2_ (ms) | 62.5 ± 4.0 | 67.9 ± 5.0 | 59.9 ± 2.5 | 331.5 ± 29.5 ^****^ |
| _%_  fast | 46.3 ± 2.9 | 43.6 ± 3.4 | 47.6 ± 1.8 | 24.8 ± 1.7^****^ |
| n | 18 | 15 | 15 | 21 |

Data given as mean ± SEM (n = number of oocytes). ^*^p ≤ 0.05, ^**^p ≤ 0.01, ^***^p ≤ 0.001,

^****^p ≤ 0.0001.
